# P-115. AI-Enhanced Complication Prediction in Brain Abscess: Validation of Predictive Clinical Parameters in a Pilot Study

**DOI:** 10.1093/ofid/ofaf695.343

**Published:** 2026-01-11

**Authors:** Amir Akhavan, Victoria Dunson, Swapan Nath

**Affiliations:** TCU Burnett School of Medicine, Fort Worth, Texas; TCU Burnett School of Medicine, Fort Worth, Texas; TCU Burnett School of Medicine, Fort Worth, Texas

## Abstract

**Background:**

Brain abscess can present as a stroke mimic, making early diagnosis challenging. Prompt identification of patients at risk for neurologic deterioration is critical to prevent herniation or death. Advanced AI (large language model, LLM) analytics can integrate clinical, laboratory, and imaging data to generate real-time risk profiles. We aimed to develop an AI-driven risk stratification tool for brain abscess complications.

**Methods:**

We mined the literature from the past 15 years using ChatGPT-4.5 Deep Research to extract validated predictors of neurologic decline in brain abscess (127 studies). Key factors identified informed a composite Neurologic Deterioration in Brain Abscess Score (NDBAS) incorporating Glasgow Coma Scale (GCS), abscess size, edema severity, lesion location, number of lesions, comorbidities (e.g., diabetes), and pathogen type. Structured data from an index case (60-year-old male with diabetes; serial exams, labs, and MRI findings) were applied to calculate NDBAS and assess predicted risk.

**Results:**

NDBAS highlighted high-impact predictors consistent with established risk criteria (e.g., depressed GCS, large or multiple abscesses, severe edema/midline shift). Applied to the representative case (60-year-old man presenting with right hemiplegia and a left frontoparietal rim-enhancing abscess on MRI), the score was 5, indicating moderate risk and corresponding with the need for urgent neurosurgical intervention. The model’s prediction aligned with the patient’s actual clinical course, demonstrating qualitative concordance between predicted risk and outcome.

**Conclusion:**

AI-enhanced decision-support tools like NDBAS show promise for early complication prediction after imaging confirmation of brain abscess. This pilot case illustrates potential clinical utility in guiding timely intervention. Larger-scale validation studies are recommended to further evaluate and refine the model.

**Disclosures:**

All Authors: No reported disclosures

